# Assessing changes in knowledge, attitude and practices on dengue diagnosis and management among primary care physicians after the largest dengue epidemic in Singapore

**DOI:** 10.1186/s12879-017-2525-3

**Published:** 2017-06-15

**Authors:** Junxiong Pang, Zoe Jane-Lara Hildon, Tun Linn Thein, Jing Jin, Yee Sin Leo

**Affiliations:** 1 Institute of Infectious Diseases and Epidemiology, Tan Tock Seng Hospital, 144 Moulmein Road, Singapore, 308089 Singapore; 2 Saw Swee Hock School of Public Health, National University of Singapore, Singapore, Singapore; 3 Johns Hopkins Center for Communication Programs, Johns Hopkins University, Baltimore, USA; 4 Department of Global Health and Development, Faculty of Public Health & Policy, London School of Hygiene and Tropical Medicine, London, UK; 5 Yong Loo Lin School of Medicine, National University of Singapore, Singapore, Singapore; 6 Lee Kong Chian School of Medicine, Nanyang Technological University, Singapore, Singapore

**Keywords:** Dengue epidemic, Primary care, Dengue management, Dengue diagnosis, Knowledge, Attitude, Practices

## Abstract

**Background:**

Dengue results in high morbidity and mortality globally. The knowledge, attitude and practices (KAP) of dengue management, including diagnosis, among primary care physicians (PCPs) are important to reduce dengue transmission and burden. However, there is a lack of understanding on the impact of dengue epidemic on dengue management. Hence, the aim of this study is to examine the changes in KAP on dengue management among PCPs before and after the largest dengue epidemic in 2013 in Singapore.

**Methods:**

Surveys were mailed to 2000 and 1514 PCPs registered under the Singapore Medical Council in March of year 2011 and 2014, respectively. Survey data were then collected between April and June of that year. Chi-square or Fisher’s exact test was used for comparing categorical variables. A multivariate logistic regression model was implemented to determine independent factors for frequent use of dengue diagnostic tests (DDTs). All tests were conducted at 5% level of significance. Adjusted odds ratio and corresponding 95% confidence intervals were reported, where applicable. Qualitative data were descriptively coded for themes and analysis.

**Results:**

Among PCPs surveyed in 2011 and 2014, 89.9% and 86% had good knowledge on dengue management respectively. The usage of DDTs had increased significantly in 2014 (*N* = 164;56%) as compared to 2011 (*N* = 107;29.5%) in both private and public clinics (*p* < 0.001). Dengue Duo point-of-care test (POCT) kits was independently associated with frequent use of DDTs (adjusted odds ratio = 2.15; 95% confidence interval = 1.25–3.69). There was a significant reduction in referral of dengue patients to hospital (31.4% in 2011; 13.3% in 2014; *p* < 0.001), and a significant increase in frequency of clinic follow-ups (18.4% in 2011; 28.5% in 2014; *p* = 0.003). One key theme highlighted was that dengue management can be improved with availability of POCT kit, better awareness of the disease and any revised clinical guidelines.

**Conclusion:**

The knowledge on dengue management remained high, while the attitude and practices, particularly on the usage of DDTs improved significantly after a large epidemic. Furthermore, PCPs had more confident in managing dengue patients in primary care settings and in educating patients on the importance of vector control and dengue warning signs to reduce dengue transmission and burden.

**Electronic supplementary material:**

The online version of this article (doi:10.1186/s12879-017-2525-3) contains supplementary material, which is available to authorized users.

## Background

Dengue, one of the major global burdens, is estimated to affect at least 50 million people annually [1]. Dengue haemorrhagic fever (DHF) and Dengue shock syndrome (DSS) are severe clinical manifestations for dengue infection in both adult and children. Identifying the disease early and predicting the possible outcomes effectively is a leading concern for Public Health specialists managing the disease [2]

Dengue fever (DF) is endemic in the tropical and sub-tropical regions, including Singapore [2]. Dengue was first reported in Singapore in the early 1960 [3]. Since 1969, with the implementation of vector control and surveillance, public health education and law enforcement, there has been a general decline in disease incidence, until in 1990 when dengue began to surge again [4]. Since then, Singapore has faced several dengue epidemic. Spikes have been recorded in 2005 and 2007 with 14,209 and 8826 cases respectively [5]. The largest dengue epidemic was experienced in the year 2013 with 22,170 reported dengue cases with eight deaths [6]. In Singapore, the occurrence of DF and DHF are influenced by several factors such as lowered herd immunity and virus transmission outside the home by *Aedes* mosquitoes, which potentially resulted in the resurgence of dengue incidences among adult population [7].

In 1997, the World Health Organization introduced dengue management guidelines for diagnosis, treatment, prevention and control [8]. A revised edition was released in 2009 with updated information for better clinical management, vector management and laboratory diagnosis [9]. Dengue virus infection may be asymptomatic or symptomatic that may lead to unpredictable severe outcomes such as death. In the three largest outbreak clusters in Singapore, it was revealed that 73.2% of residents with recent infection were asymptomatic [5]. Hence, early detection of these dengue cases and monitoring of disease severity are important parts of dengue management to reduce dengue transmission and burden.

Moreover, to avoid over-hospitalization during an epidemic, it is important to have a system not only for early detection but also effective outpatient monitoring. Since the development of Dengue Duo POCT kit [10], there were many clinical validation studies performed such as the SD Dengue Duo POCT kit (Standard Diagnostics, Inc., Gyeonggi-do, Korea) with sensitivities and specificities ranging from 75.5–92.9% and 88.8–100% respectively using frozen serum or plasma samples [11–14]. This commercially available assay uses whole blood to obtain dengue positivity result within 30 min of patient presentation. In 2013, SD Dengue Duo POCT kit was also validated in Singapore with sensitivity and specificity of 93.9% and 92.0% respectively [15], with significant reduction (from estimated four hours to an hour) in the time required from patient presentation to appropriate clinical management in a tertiary hospital. This kit, which includes both dengue NS1 antigen and dengue IgM & IgG antibody detection, is likely to enhance early diagnosis of dengue and reduce dengue burden at the primary healthcare settings. A good understanding of the changes in KAP of the dengue management among the PCPs will be important to reduce dengue burden. Even though there were two cross-sectional KAP studies focusing on the dengue management of PCPs in Southern Vietnam [16] and Southern Taiwan [17], there is still a lack of understanding on the changes of KAP after a large dengue epidemic.

Therefore the aims of this study were to (1) Examine changes in primary care physicians knowledge, attitude and practices, in particular on the usage of dengue diagnostic tests and related clinical practices; (2) Enquire about their experience on using the POCT kit; (3) Gather recommendations for improved service delivery in patient diagnostics and clinical care management to facilitate reduction in dengue burden.

## Methods

### Study design

Surveys were mailed to 2000 and 1514 PCPs registered under the Singapore Medical Council in March of year 2011 [18] and 2014, respectively. Survey data were then collected back between April and June of that year. The survey form was developed by clinical researchers and was piloted prior to the actual survey. A total of 364 (18.3%) and 293 (19.4%) surveys were mailed back in 2011 and 2014, respectively. We compared survey data using baseline data collected in 2011 [18] and a follow-up survey in 2014. The survey included open-ended questions enquiring about the usage experience and/or perception of the new POCT kit introduced in 2014 to facilitate dengue diagnosis.

### Survey questionnaire

The questionnaire was designed with four parts. They were (1) practitioner demographics (gender, age, type of practice and qualification), (2) dengue management – including related knowledge, dengue diagnostic practices, and clinical care; (3) attitudes toward POCT and (4) responses to the dengue epidemic in 2013. The baseline survey included 25 multiple-choice questionnaires [18]. However, only 18 of these questions were retained in the 2014 survey so that meaningful comparisons focusing on dengue management can be performed (Additional file 1).

In relation to repertoires of disease management, participants were asked about their preferred method of dengue diagnosis, the level clinical care of confirmed patients, and their knowledge on dengue clinical guidelines. The dengue warning signs recommended by the World Health Organization used to classify Dengue into levels of severity was assessed [1]. These warning signs include: abdominal pain or tenderness, persistent vomiting, clinical fluid accumulation, bleeding/mucosal bleeding, drowsiness, fatigue, lethargy, breathlessness, increase in haematocrit concurrent with rapid decrease in platelet count and hepatomegaly. Participants were asked about their awareness of the warning signs and to list down three common ones.

In the follow-up survey in 2014, participants were asked about their awareness on rapid Dengue POCT, their level of interest in using it and any perceived challenges/benefits if they have experience using it. Two open-ended questions were designed to seek participants’ opinion and experience about surge management, and how participants had managed the 2013 epidemic and the changes in dengue management after these dengue epidemics.

### Analytic methods

Analysis was performed with consideration of both the quantitative and qualitative data gathered in the survey. For the quantitative analyses, the data were stratified by type of practice, i.e. government subsidized polyclinic, private practice. Chi-square or Fisher’s exact test was used for comparing categorical variables. A multivariate logistic regression model was implemented to determine independent factors for frequent use of DDTs. All tests were conducted at 5% level of significance. We reported adjusted odds ratio and corresponding 95% confidence intervals where applicable. All statistical analyses were performed using SPSS Version 16 (SPSS Inc., Chicago, IL). Qualitative data were descriptively coded for themes on the topics of the experience of using the POCT kit, the management of dengue epidemics and changes in dengue management after dengue epidemic. NVivo 10 software Version 10, 2012 (QSR International Pty Ltd.) was used to manage the textual data.

## Results

### Demographics characteristics of PCPs surveyed in 2011 and 2014

A total of 364 PCPs participated (18.3% response rate) in the survey in 2011 [18]. A total of 293 PCPs participated (19.4% response rate) in the survey in 2014 (Table 1). Among these, majority were male (*N* = 157; 53.6%), and were pre-dominantly in the age-group of 41–60 years old (*N* = 185; 63.1%) and from the private sector (*N* = 206; 70.3%). There was significantly lesser male PCPs (*p* = 0.046) in year 2014 (*N* = 157; 53.6%) than year 2011 (*N* = 223; 61.3%). There were significantly more PCPs in the 41–60 years old group, but lesser in the above 60 years old group (*p* < 0.0001) in the survey year 2014 (*N* = 185; 63.1% & *N* = 26; 8.9% respectively) than year 2011 (*N* = 185; 50.8% & *N* = 67; 18.4% respectively). The proportion of PCPs with post-graduate degree qualifications was significantly higher in survey year 2014 (*N* = 158; 53.9%) than in 2011 (*N* = 124; 34.1%; *p* < 0.001; Table 1).

**Table 1:** Demographics of primary care physicians

Years	2011 *N* = 364 (%)	2014 *N* = 293 (%)	*P* value	CFPS 2011^a^ *N* = 1400 (%)
Gender				
Male	223 (61.3)	157 (53.6)	**0.046**	(60)
D.N.P.	3 (0.8)	1 (0.3)		
Age				
21–40 years	111 (30.5)	82 (28.0)		(58) for <45 yrs
41–60 years	185 (50.8)	185 (63.1)		
61 years or above	67 (18.4)	26 (8.9)	**<0.001**	(42) for ≥45 yrs
D.N.P.	1 (0.3)	0		
Highest Qualifications				
M.B.,B.S. (or equivalent) only	231 (63.5)	133 (45.4)		N.A.
Post-graduate degrees	124 (34.1)	158 (53.9)	**<0.001**	N.A.
D.N.P.	9 (2.4)	2 (0.7)		
Practice				
Private	253 (69.5)	206 (70.3)		(55.9)
Polyclinic	105 (28.8)	87 (29.7)	0.931	(44.1)
D.N.P.	6 (1.7)	0		

Post-graduate degrees- Graduate Diploma in Family Medicine, Master in Medicine (Family Medicine), Membership of Royal College of Physicians

*P* values calculated by Fisher’s exact test. *P *value in bold represents a statistical significant difference

*D.N.P.* Data not provided by participants, *M.B.B.S.* Bachelor of Medicine and Bachelor of Surgery, *N.A.* Not available

^a^ *CFPS* College of Family Physicians Singapore (As of June 2011) [24]

### Knowledge on dengue warning signs and clinical management

Majority of PCPs (*N* = 250; 86%) knew at least one of the World Health Organization-guided dengue warning signs in survey year 2014, which is significantly higher than in year 2011 survey (*N* = 285; 78.3%; *p* = 0.024) (Table 2). The top three responses in survey year of 2014 include the bleeding/mucosal bleeding (*N* = 139; 55.6%) followed by abdominal pain or tenderness (*N* = 97; 38.8%) and drowsiness, fatigue, lethargy, breathlessness (*N* = 44; 17.6%) (Additional file 2). Based on the three clinical scenarios designed to assess the level of clinical management, the majority of PCPs (75.7%) gave the preferred responses in at least 2 questions (Additional file 3). In overall, 89.9% and 86% of the PCPs surveyed had good knowledge on dengue management in 2011 and 2014 (*p* > 0.05), respectively.

**Table 2:** Dengue diagnostic practices among primary care physicians

	Overall		*P* value	Private practice		*P* value	Polyclinic practice		*P* value
	2011 *N* = 364 (%)	2014 *N* = 293 (%)		2011 *N* = 253 (%)	2014 *N* = 206 (%)		2011 *N* = 105 (%)	2014 *N* = 87 (%)	
WHO warning signs									
Yes	285 (78.3)	250 (85.3)	**0.024**	N.A.	171 (83.4)	N.A.	N.A.	79 (91.9)	N.A.
D.N.P.	3 (0.8)	2 (0.7)			2 (1)			1 (1.1)	
Dengue Diagnostic Test (DDT)									
Always (100%)	107 (29.4)	164 (56.0)	**<0.001**	98 (38.7)	111 (53.9)	**0.001**	7 (6.7)	53 (60.9)	**<0.001**
Often (51–99%)	69 (19.0)	72 (24.5)	0.086	51 (20.2)	52 (25.2)	0.216	17 (16.2)	20 (23.0)	0.274
Sometimes (1–50%)	105 (28.8)	40 (13.7)	**<0.001**	61 (24.1)	26 (12.6)	**0.002**	41 (39)	14 (16.1)	**<0.001**
Never (0%)	82 (22.5)	17 (5.8)	**<0.001**	43 (17.0)	17 (8.3)	**0.008**	39 (37.1)	0	**<0.001**
D.N.P.	1 (0.3)	0		0	0		1 (1)	0	
Dengue Diagnostic Test (DDT)									
> 50% of the time	176 (48.4)	236 (80.5)	**<0.001**	149 (58.9)	163 (79.1)	**<0.001**	24 (22.9)	73 (83.9)	**<0.001**
D.N.P.	1 (0.3)	0		0	0		1 (1)	0	
Most Frequently Used DDT Among Those Who Reported Usage of DDT									
Dengue serology (IgM/IgG)	211 (74.8)	91 (33)	**<0.001**	144 (68.6)	59 (31.2)	**<0.001**	62 (93.9)	32 (36.8)	**<0.001**
Dengue non-structural antigen 1 (NS1) assay	31 (11)	62 (22.5)	**<0.001**	29 (13.8)	47 (24.9)	**0.003**	1 (1.5)	15 (17.2)	**0.002**
Dengue RT-PCR	37 (13.1)	9 (3.3)	**<0.001**	36 (17.1)	7 (3.7)	**<0.001**	1 (1.5)	2 (2.3)	>0.999
Dengue duo POCT kit	0 (0.0)	104 (37.7)	**<0.001**	0	67 (35.4)	**<0.001**	0	37 (42.5)	**<0.001**
D.N.P.	3 (1.1)	10 (3.5)		1 (0.5)	9 (4.8)		2 (3)	1 (1.2)	

*D.N.P.* Data not provided by participants, *RT-PCR* Reverse-transcription polymerase chain reaction, *N.A.* Not available

*P* value in bold represents a statistical significant difference

### Attitude and practices on dengue diagnostic tests

The proportion of the PCPs who always use DDTs, instead of relying only on clinical diagnosis, increased significantly in survey year 2014 (*N* = 164; 56%) compared to 2011 (*N* = 107; 29.5%; *p* < 0.001; Table 2). The proportion of public PCPs who always use DDTs increased significantly in survey year 2014 (*N* = 53; 60.9%) compared to 2011 (*N* = 9; 7.9%; *p* < 0.001). Moreover, the proportion of public (0% in 2014 and 36.8% in 2011; *p* < 0.001) and private (8.3% in 2014 and 17% in 2011; *p* = 0.008) PCPs who had never used diagnostic testing decreased significantly in survey year 2014 compared to survey year 2011. A significantly higher proportion of PCPs (*N* = 236; 80.5%) used DDTs in more than 50% of their consultations for suspected dengue in survey year 2014 compared to year 2011 (*N* = 176; 48.5%; *p* < 0.001).

In 2011, about 74.8% (*N* = 211) of the PCPs relied on Dengue serology (IgM/IgG) test in the laboratory as Dengue Duo POCT kit was not available in the market then. In 2014, after the introduction of Dengue Duo POCT kit, about 37.7% (*N* = 104) of the PCPs used the Dengue Duo POCT kit. Besides the Dengue Duo kit, Dengue serology (IgM/IgG) test and Dengue non-structural antigen 1 assay test were frequently used in 2014 (*N* = 91, 33% and *N* = 62, 22.5% respectively). The proportion of PCPs who used dengue reverse transcription- polymerase chain reaction assay was significantly reduced in survey year 2014 (*N* = 9, 3.3%) compared to survey year 2011 (*N* = 37, 13.1%; *p* < 0.001). These trends are similar in both private and public polyclinics (Table 2).

Using data from 2014 survey, a multivariate logistic regression was performed to identify factors associated with frequent behaviour (comparing between ‘Always’ and ‘Sometimes/Never’ groups) of DDTs utilization. After adjusting for confounding factors (gender, age, place of practice and qualifications), only Dengue duo POCT kits was found to be independently associated with frequent use of DDTs (adjusted odds ratio = 2.15, 95% confidence interval = 1.25–3.69) (Table 3).

**Table 3:** Factors associated with ‘always’ versus ‘often/sometimes’ behaviour of using Dengue diagnostic test in survey year 2014^a^

Variables in equation	Adjusted Odds Ratio (95% confidence interval)	*P* value
Type of Dengue diagnostic test		
Other tests	Referent	
Dengue duo POCT kit	**2.15 (1.25–3.69)**	**0.01**
Gender		
Male	Referent	
Female	1.16 (0.69–1.96)	0.57
Age		
21–40 years	Referent	
41–60 years	0.71 (0.38–1.32)	0.27
61 years or above	0.72 (0.24–2.14)	0.56
Place of practice		
Private	Referent	
Polyclinic	0.79 (0.42–1.46)	0.45
Qualifications		
M.B.,B.S. (or equivalent)	Referent	
GDFM	1.05 (0.56–1.95)	0.88
M.Med (Fam. Med.)	1.08 (0.56–2.10)	0.81
MRCP	2.81 (0.30–26.62)	0.37

*P* values calculated by binary logistic regression. *P* value in bold represents a statistical significant association﻿

*GDFM* Graduate Diploma in Family Medicine, *M.Med (Fam. Med.)* Master in Medicine (Family Medicine), *MRCP* Membership of Royal College of Physicians

^a^2014 study only

### Clinical care and practices for dengue

Compared with survey year 2011, significantly smaller proportion of PCPs referred more than 50% of their dengue patients to hospital in survey year 2014 (*N* = 114, 31.4% in 2011 vs. *N* = 39, 13.3% in 2014; *p* < 0.001; Table 4). About 45.4% of the PCPs set the platelet count of ≤80,000/mm^3^ as cut off point for referral to hospital, although a significant increase was also seen in proportion of PCPs who uses the platelet count of ≤50,000/mm^3^ (*N* = 83, 22.8% in 2011 vs *N* = 115, 39.2% in 2014; *p* < 0.001). Moreover, there was a significant increase in proportion of PCPs who reviewed dengue patients on average 5–6 times as part of the dengue management in survey year 2014 (*N* = 83, 28.3%) as compared to survey year 2011 (*N* = 66, 18.1%; *p* = 0.003). This trend is similar among private and public PCPs. About 57–59% of the PCPs monitored the fluid intake/urine output more than 50% of their time during patient management in both survey years. Within the public polyclinics, there was a significant increase in proportion of PCPs in survey year 2014 (*N* = 59, 67.8%) who monitored the fluid intake/urine output more than 50% of their time during patient management compared to survey year 2011 (*N* = 61, 58.1%; *p* < 0.001). There was not much difference in the proportion of PCPs who performed postural blood pressure monitoring more than 50% of their time during patient management in general. However, there was a significant increase (*N* = 61, 58.1% in 2011 vs *N* = 59, 67.8% in 2014; *p* < 0.001) in proportion of PCPs who performed postural blood pressure monitoring more than 50% of their time during patient management in the public PCPs, but a significant reduction among private PCPs (*N* = 126, 49.8% in 2011 vs *N* = 82, 39.8% in 2014; *p* = 0.038; Table 4).

**Table 4:** Dengue clinical management practices among primary care physicians

	Overall		*P* value	Private practice		*P* value	Polyclinic practice		*P* value
	2011 *N* = 364 (%)	2014 *N* = 293 (%)		2011 *N* = 253 (%)	2014 *N* = 206 (%)		2011 *N* = 105 (%)	2014 *N* = 87 (%)	
Referral to hospital									
> 50%	114 (31.4)	39 (13.3)	**<0.001**	99 (39.1)	36 (17.5)	**<0.001**	14 (13.3)	3 (3.4)	**0.020**
D.N.P.	1 (0.3)	0		1 (0.4)	0		0	0	
Usage of platelet count									
≤ 100,000/mm3	88 (24.2)	30 (10.2)	**<0.001**	79 (31.2)	29 (14.1)	**<0.001**	8 (7.6)	1 (1.1)	**0.042**
≤ 80,000/mm3	190 (52.2)	133 (45.4)	0.085	117 (46.2)	101 (49.0)	0.574	70 (66.7)	32 (36.8)	**<0.001**
≤ 50,000/mm3	83 (22.8)	115 (39.2)	**<0.001**	54 (21.3)	66 (32.0)	**0.010**	27 (25.7)	49 (56.3)	**<0.001**
Platelet count is not an indicator	3 (0.8)	15 (5.1)	**0.001**	3 (1.2)	10 (4.9)	0.230	0 (0.0)	5 (5.7)	**0.018**
Average number of clinical review per patient									
1–2	64 (17.6)	29 (9.9)	**0.005**	49 (19.4)	20 (9.7)	**0.004**	14 (13.3)	9 (10.3)	0.656
3–4	217 (59.6)	171 (58.4)	0.688	140 (55.3)	118 (57.3)	0.775	74 (70.5)	53 (61)	0.172
5–6	66 (18.1)	83 (28.3)	**0.003**	48 (19)	59 (28.6)	**0.020**	16 (15.2)	24 (27.6)	**0.049**
> 6	12 (3.3)	8 (2.7)	0.820	11 (4.3)	7 (3.4)	0.637	1 (1)	1 (1.1)	1.000
D.N.P.	5 (1.4)	2 (0.7)		5 (2)	2 (1)		0	0	
Fluid Intake/Urine Output Test									
> 50% of the time	208 (57.1)	172 (58.7)	0.751	142 (56.1)	113 (54.9)	0.777	61 (58.1)	59 (67.8)	**<0.001**
D.N.P.	1 (0.3)	0		0	0		1 (1)	0	
Use of postural blood pressure									
> 50% of the time	179 (49.2)	121 (41.3)	**0.049**	126 (49.8)	82 (39.8)	**0.038**	61 (58.1)	59 (67.8)	**<0.001**

*D.N.P.* Data not provided by participants, *RT-PCR* reverse-transcription polymerase chain reaction, *NA* not applicable

*P *value in bold represents a statistical significant difference

### Knowledge, attitude and experience toward POCT kits

About 68.9% (*N* = 202) of the PCPs in survey year 2014 heard about POCT, and 88.1% (*N* = 258) of the PCPs were interested to know more about POCT (Table 5). The top three preferred methods of gaining more information was through postal information (*N* = 76, 31.5%), website with video (*N* = 70, 29%) and through email and written documentation (*N* = 68, 28.2%).

**Table 5:** Knowledge on point of care rapid Dengue test based on survey performed in year 2014 (*N* = 293)

	N (%)
Heard about point of care rapid Dengue test	
Yes	202 (68.9)
No, Not sure	89 (30.4)
D.N.P.	2 (0.7)
Wish to know more about point of care rapid Dengue test	
Yes	258 (88.1)
No	31 (10.6)
D.N.P.	4 (1.3)
Preferred mode of information^a^	
Postal written information	76 (31.5)
Website with video	70 (29.0)
Email and written demonstration	68 (28.2)
Seminar through demo	41 (17.0)
Public posters	8 (3.3)
Other	4 (1.7)

*D.N.P.* Data not provided by participants

^a^Multiple an**s**wered allowed

Only 41 (20.3%) out of 202 PCPs who have heard about POCT had personally experienced using POCT (Table 6), even though 104 PCPs (Table 2) had reported using POCT as their preferred method for diagnosis of dengue. The top three perceived benefits in using POCT in their clinic were saving patient’s waiting time (*N* = 203, 84.2%), helping case management (*N* = 195, 80.9%) and more accurate diagnosis (*N* = 137, 56.8). In addition, the top three perceived challenges in using POCT in their clinic were “not cost-effective” (*N* = 61, 25.3%), “existing heavy workload” (*N* = 41, 17%) and “no time for training” (*N* = 32, 13.3%).

**Table 6:** Usage on point of care rapid dengue test

	N (%)
Usage of point of care rapid dengue test in clinic among PCPs who knows about it (*N* = 202)	
Yes	41 (20.3)
No	157 (77.7)
D.N.P.	4 (2)
Duration of using point of care rapid dengue test (*N* = 41)	
< 1 month	4 (9.8)
1–3 months	3 (7.2)
> 3 months	30 (73.2)
D.N.P.	4 (9.8)
Benefits perceived by PCPs in using POCT in their clinic^a^	
Saves waiting time	203 (84.2)
Helps case management	195 (80.9)
More accurate diagnosis	137 (56.8)
Minimum usage of resources	91 (37.8)
Manage surge in Dengue	87 (36.1)
Don’t know about it	44 (18.3)
Others (benefits)	8 (3.3)
Challenges perceived by PCPs in using POCT in their clinic^a^	
Not cost effective	61 (25.3)
Too much workload	41 (17.0)
Too much time for training	32 (13.3)
Not accurate	27 (11.2)
No challenges	26 (10.)
No comment	1 (0.4)
Others (Challenges)	49 (20.3)

*D.N.P.* Data not provided by participants

^a^Multiple answered allowed

The attitude and perception of PCPs were sought in a qualitatively method on their current usage and implementation of POCT in the clinic using open ended questions. Based on the responses received among those PCPs who are currently using POCT, the common reasons for usage included “the availability of Dengue duo POCT kit in their clinic” and “it is a standard test in their clinic” (Table 7). Other advantages that were highlighted were “the flexibility to diagnose in both early and late phase of fever”, “easy to use” and “having accurate result”. In general, they felt that POCT is useful and two of the PCPs who responded also highlighted that “patients really appreciate a quick diagnosis”. However, there was also some PCPs who expressed concerns, despite the current usage of POCT. They expressed concern about the cost and accuracy in using POCT as well as the fact that laboratory tests are likely more accurate than POCT kit.

**Table 7:** Positive and negative experiences among PCPs who are using or have used POCT before

Themes	Frequency	Quotes
Positive		
Can be used to diagnose in both early and late phase of fever	36	*“NS1 can detect Dengue before D5 fever. IgM/IgG for fever > D5.”*
Available	21	*“Available in our clinic.”* *“This is usually what’s available at our lab.”*
Good/Useful	16	*“Best thing since sliced bread”* *“Useful as a good screening tool when Dengue fever is suspected”*
Free	15	*“Free test* via *EHI diagnosis”*
Standard test	14	*“Standard protocol.”* *“Standard test at commercial lab used.”*
Rapid result	10	*“I love using it. Gives rapid diagnosis on Dengue fever suspect”*
Accurate result	5	*“Dengue duo- quite happy with it so far accurate.”*
Easy to use	5	*“Using it seems simple enough.”*
Diagnosis in early phase of fever	2	*“Very useful for early identification of cases so patients are more likely to agree to do daily blood for monitoring.”*
Patient’s positive opinion	2	*“Patients really appreciate a quick diagnosis.”*
Cost-effective	1	*“I find it useful, fast selective and cost effective.”*
Negative		
Concern about cost	9	*“Given free so used it for trial otherwise expensive”*
Concern about accuracy	7	*“After doing 3 cases on suspected Dengue- had 2 false negative- not found!”*
Troublesome to use	2	*“Cumbersome / troublesome to use.”*
Trust	2	*“I only use it at night or Sunday... Still trust the lab test for Dengue more than portable kit.”*
Other test also used	2	*“Has to supplemented by FBC sent to the lab to have haematocrit/platelet.”*
Not had occasion to use it much	2	*“[used] very little [so] not enough suspected cases seen.”*
Only used when instructed to	1	*“Have used it during referral to a study in our clinic. But now stopped.”*
Lack of official evaluation of it’s efficacy	1	*“Lack of official reports.”*
Patient’s negative opinion	1	*“Patients are still new to the idea of a rapid test kit.”*

Among PCPs who have not used POCT in the clinic, they had the perception that POCT has “very little improvement in patients’ management”, and the current overwhelming workload of their staffs actually deterred them from using (Additional file 4: Table S1). A major concern for private PCPs was the cost of expired kits. For instance, expired kits will be a loss for their business if there are not enough suspected dengue patients for testing. The lack of an official test report for their patients’ assurance is also one of the reason of not using POCT. Moreover, it was highlighted that the Environmental Health Institute in Singapore provides free laboratory dengue testing for interested PCPs who are keen to participate in their dengue surveillance program so there is no urgency in trying out these POCTs. Despite these concerns, some PCPs anticipated improvements in efficiency of patient diagnosis after implementing POCT, so they were keen to use POCT in the near future.

### Perception of surge management during dengue largest spike in 2013

There were two final open-ended questions that focused on the experience of PCPs during the largest dengue spike in 2013 (Additional file 4: Table S2) as well as the changes in disease management since the outbreaks in 2005 and 2007 (Additional file 4: Table S3). In general, physicians claimed to be more vigilant, ordered full blood count and dengue diagnosis tests for suspected patients more frequently than before. Although there was a dengue spike in 2013, 38 of the PCPs reflected that the situation was still manageable in their clinics. For confirmed cases, they did close monitoring on full blood count and other symptoms more frequently. They also played a more active role in educating patients on dengue warning signs and the importance of vector control (Additional file 4: Table S2).

There were 187 PCPs who had practiced during the earlier smaller dengue spikes in 2004 and 2007. The majority of them felt that case management has since improved. Majority attributed to the availability of better dengue diagnosis tests, as well as better awareness of the disease among the community and doctors. Moreover, clearer clinical guidelines were also made more available and accessible to PCPs since then. The PCPs also highlighted that doctors were more confident in managing dengue patients as outpatients and so fewer patients were referred to hospital (Additional file 4: Table S3).

## Discussion

Primary care physicians (PCPs) play very important role in early diagnosis and notification of dengue cases to reduce dengue transmission and burden [1]. The right knowledge, attitude and practices on dengue management among PCPs are critical for dengue control and public education [16, 17]. Since 2011, there have been advances in providing better dengue management for suspected dengue patients in Singapore. Gan et al. in 2014 reported the high performance of point-of-care diagnostic Dengue duo test in detecting dengue infection in a clinic setting in Singapore [15]. Leo et al. in 2013 reported the validation of the new World Health Organization guideline in Singapore. The study reported that dengue patients without any of the seven warning signs as stated in the guideline may be managed as outpatients, while referral of patients who presented with any of these warning signs may lead to over-hospitalization [19].

Over the years between year 2011 and 2014, the proportion of PCPs who always diagnosed dengue using diagnostic test had increased by about two folds, and those who never used diagnostic test to confirm dengue fell by about three folds (Table 3). Compared to private PCPs, all of PCPs from polyclinics had higher preference to perform diagnostic test for patients with suspected dengue in 2014. Although Dengue duo POCT kit was only introduced in 2013, more than one third of PCPs had preference to use the POCT kit based on the survey in 2014. On further assessment, Dengue duo POCT test was independently associated with frequent use of diagnostic test after adjusting for age, gender, level of professional qualification and place of practice. This highlights the impact of POCT in increasing the usage of diagnostic test in primary healthcare setting to confirm dengue for early management of dengue cases as well as for dengue control via notification to Ministry of Health.

The PCPs emphasized that availability of the POCT kit at the place of practice, accuracy of the test, rapidness of results and cost-effectiveness favored its use. The PCPs’ preference to receive more information on diagnostic tests was through postal mails or website with video demonstration on its usage. They were also concerns over the use of this new POCT kit. These concerns were the lack of quality control and increased workload in the clinic, which were also highlighted in another study by Pai et al. in 2012 [20]. Our findings suggested that further development of more accurate and user-friendly test kits, as well as having highly accessible training platforms for front line care providers are likely to increase the use of dengue diagnostic test in both public and private primary healthcare settings to enhance patient management.

After the large dengue epidemic, most PCPs felt that dengue case management had improved. This was likely due to the availability of better dengue diagnostic tests, greater awareness of the disease and clearer clinical guidelines. Compared to the 2011 survey, the survey in 2014 showed PCPs referred patients to hospital with a lower platelet cut-off of ≤50,000/mm^3^, which is clinically more relevant for close monitoring. Similar platelet cut-off was also reported by Thaver et al. in 2011 amongst the Pakistanis PCPs [21]. We also found that our Singapore PCPs became more confident, and committed to managing the dengue patients as outpatients, with closer monitoring, where possible. This was reflected in the significant reduction in patient referrals to hospital and the increased number of clinical reviews at the primary healthcare setting.

Carrasco et al. in 2011 highlighted that managing dengue cases in hospital incurred 10 times more cost than ambulatory care in Singapore ($600 vs. $60 per day, respectively) [22]. Lee et al. also reported in 2013 that after the implementation of the new admission criteria including dengue outcome calculator which utilizes simple and readily available clinical information, the proportion of dengue cases who were being managed as inpatients fell from 91.9% in 2006 to 53.9% in 2008 with saving of US$ 1.4 million in year 2008 [23]. Similar cost savings to both patients and the government is likely possible with a significant reduction in dengue referrals to hospital with the increasing usage of POCT based on the findings in this study.

We were limited by the fact that there were significant differences in gender, age groups and the highest qualifications attained among the PCPs surveyed in 2011 and 2014. As such, we cannot completely exclude the impact of these demographic differences. However, regardless of the demographic differences, the knowledge level on clinical management remained high, suggesting that the impact on PCPs’ attitude and practices of dengue diagnosis should be relatively small. We were also limited by the survey method in collecting more explorative qualitative data. However, open-ended survey questions provided additional in depth information about physicians attitudes and experiences on dengue clinical management and diagnosis. The case management data that was collected was based on three case management scenarios (Additional file 3) with the aim to understand common practices amongst primary care physicians. These data will not be able to capture the optimal case management of a suspected dengue case but only the current preferred case management of a suspected dengue case. In fact, Dengue has many overlapping features with other febrile illnesses. It would be ideal to apply simple, cheap and easy to use diagnostic test at primary care sites for optimal case management. In addition, this study involved only PCPs who were practicing during the 2013 epidemic due to their valid practicing licenses and clinic addresses. As such, this study is not able to conclude if PCPs who were not practicing during 2013 epidemic had the same awareness as those PCPs who were practicing. Last but not least, the low response rate may not provide a complete understanding of the changes in KAP on dengue management after a large dengue epidemic. However, a comparison on the demographics of PCPs between this study and another national study published in 2014 [24], the proportion of PCPs surveyed reflects the general PCPs reasonably well, although there are more older PCPs than younger PCPs (<45 years old) in both 2011 and 2014 surveys (Table 1).

## Conclusions

After the largest dengue outbreak in 2013, knowledge on clinical management of dengue remain high, but not higher between year 2011 and 2014. However, there were significant improvements in the diagnosis of dengue, particularly on the importance of using a diagnostic kit such as the dengue Duo POCT kit. There were also significant increase in awareness and practices of the best practices of dengue clinical management between 2011 and 2014. In order to improve acceptance & usage of the POCT kit to facilitate early diagnosis of dengue, it may be worth emphasizing the importance of continuous medical education via web-based video trainings and postal information, the sharing of formal evaluations of the diagnostic tests in local settings through seminars or dialogue sessions, as well as the support of the local government in providing subsidy for the usage of POCT kit in the primary healthcare settings.

## Additional files

Additional file 1: Survey on knowledge, attitudes, practice related to dengue management amongst primary care physicians. This provides the questions that were used to assess the KAP of the primary care physicians. (DOC 87 kb)

Additional file 2: The awareness of the dengue warning signs among primary care physicians. Data on dengue warning signs recommended by the World Health Organization that the participants are aware of. (ODT 17 kb)

Additional file 3: The clinical scenarios used to assess the level of clinical management. Data on the three clinical scenarios surveyed to assess the level of clinical management. (DOC 55 kb)

Additional file 4: Perceptions of PCPs on POCT and clinical management experience during 2013 outbreak. Data on the perceptions of PCPs who do not have any experience on POCT and the management experience and changes during and after the 2013 outbreak. (DOCX 23 kb)

## Abbreviations

DDTs: Dengue diagnostic tests
DF: Dengue fever
DHF: Dengue haemorrhagic fever
DSS: Dengue shock syndrome
KAP: Knowledge, attitude and practices
PCPs: Primary care physicians
POCT: Point-of-care-test
