# Supplementary material for: Assessing changes in knowledge, attitude and practices on dengue diagnosis and management among primary care physicians after the largest dengue epidemic in Singapore
Source: BMC Infect Dis. 2017 Jun 15;17:428. doi: 10.1186/s12879-017-2525-3 (PMC5472871; doi:10.1186/s12879-017-2525-3)
Supplement: Supplementary file 1 — Survey on knowledge, attitudes, practice related to dengue management amongst primary care physicians. This provides the questions that were used to assess the KAP of the primary care physicians. (DOC 87 kb) [file 12879_2017_2525_MOESM1_ESM.doc]

Additional File 1:

Survey on Knowledge, Attitudes, Practice (KAP) related to Dengue Management amongst Primary Care Physicians

As part of our efforts to better understand dengue under the STOP Dengue Translational Clinical Research Programme, we are following-up to find out more about your clinical management of dengue cases.

We would appreciate 10-15 minutes of your time to complete the survey.

**Please use the enclosed pre-paid envelope to mail back the questionnaires.**

Unless otherwise indicated please tick only one box throughout.

**Answer all questions and try not to skip any questions.**

Date :____/____/_____

(dd/mm/yyyy)

***General information***

1. Gender
   - Male
   - Female
2. Age
   - 21-30 years
   - 31-40 years
   - 41-50 years
   - 51-60 years
   - 61 years or above
3. Type of practice
   - Solo practice
   - Group practice
   - Freelance/Locum
   - Polyclinic
   - Other (please specify): ________________________
4. First two digits of postal code of main site of practice (if multiple locations):

|  |  |
| --- | --- |

1. Qualification
   - M.B.,B.S. (or equivalent)
   - GDFM
   - M.Med (Fam. Med.)
   - MRCP

***On Dengue Management***

1. Do you perform a dengue diagnostic test if you suspect dengue?

| - Always | (100%) |
| --- | --- |
| - Often | (51-99%) |
| - Sometimes | (1-50%) |
| - Never | (0%) |

1. Which ONE of the following dengue diagnostic tests do you most frequently use?

- Dengue serology (IgM/IgG)
- Dengue non-structural antigen 1 (NS1) assay
- Dengue reverse-transcription polymerase chain reaction (RT-PCR)
- Dengue duo kit with NS1 AND IgM/IgG tests
- I do not perform dengue test

1. Do you perform a full blood count when suspecting dengue?

| - Always | (100%) |
| --- | --- |
| - Often | (51-99%) |
| - Sometimes | (1-50%) |
| - Never | (0%) |

1. Do you monitor confirmed or suspected dengue cases on a daily basis?

| - Always | (100%) |
| --- | --- |
| - Often | (51-99%) |
| - Sometimes | (1-50%) |
| - Never | (0%) |

1. Have you heard of ‘warning signs’ in dengue?

- Yes: AND please tell us what the top three are
  - ________________________
  - ________________________
  - ________________________
- No
- Not sure

1. The full blood count of a patient at day 2 of fever revealed total white cells of 8,700/ul (Poly 75%, lymph 15%, monocyte 5%, others 5%), hemoglobin of 15g/dl. By the look of the full blood count alone, I can conclude that dengue is an unlikely diagnosis.

- Yes
- No
- Not sure

1. During the entire course of dengue, the critical period denotes the period when patients are having high fever and look ill.

- Yes
- No
- Not sure

1. The minimum and maximum hematocrit readings of a confirmed dengue patient were 42% and 51% respectively, what was the percentage change of hematocrit?

- 17.6%
- 21.4%
- 9%
- Not sure

1. Do you ask for fluid intake and/or urine output in patients with suspected or confirmed dengue?

| - Always | (100%) |
| --- | --- |
| - Often | (51-99%) |
| - Sometimes | (1-50%) |
| - Never | (0%) |

1. Do you measure postural blood pressure in patients with suspected or confirmed dengue?

| - Always | (100%) |
| --- | --- |
| - Often | (51-99%) |
| - Sometimes | (1-50%) |
| - Never | (0%) |

1. Do you perform full blood counts (FBC) to monitor patients with suspected or confirmed dengue on a daily basic?

| - Always | (100%) |
| --- | --- |
| - Often | (51-99%) |
| - Sometimes | (1-50%) |
| - Never | (0%) |

1. Please indicate the platelet count below which you would normally refer patients for hospital management:

- <100 000/mm3
- <80 000 /mm3
- <50 000 /mm3
- Platelet count is not an indicator

1. Do you find monitoring haematocrit important in patients with suspected or confirmed dengue?

- Yes
- No
- Not sure

1. Currently, what is a rough proportion of dengue patients that you would refer to the hospital?

- 0-25%
- 26-50%
- 51-75%
- 76-100%

1. On average, what is your approximate number of clinic review for the entire course of dengue follow up?

- 1-2
- 3-4
- 5-6
- >6

1. Do you routinely check for normalization of laboratory tests such as platelet count and liver enzymes after a patient has recovered from dengue?

| - Always | (100%) |
| --- | --- |
| - Often | (51-99%) |
| - Sometimes | (1-50%) |
| - Never | (0%) |

On newer practices for dengue management

1. Have you heard about a dengue diagnostic kit that can be done in the clinic with quick turnaround time, often referred to as the point of care rapid dengue test?

- Yes, AND ALSO TICK ALL that apply:
  - I heard of it through our independent medical suppliers
  - A medical colleague mentioned it to me
  - I came across it while researching this myself
  - Our government contacts had recommended its use
  - Others (please specify): ___________________________

______________________________________________

- No, I’ve never heard it mentioned before seeing it in this survey
- Not sure

1. Would you like to know more about the point of care rapid dengue test?

- Yes AND ALSO RANK 1-6 the your preferred method of learning more

(1= best and 6= least appealing)

- Postal written information
- Email and written demonstration
- Website – with video demonstration
- Seminar with taught demonstration
- Public posters for the clinical office
  - Others (please specify): ___________________________

______________________________________________

- No, it does not interest me further

1. Are you currently using a point of care rapid dengue test in your clinic?

- Yes, we have been using it in my clinic, AND TICK THE ONE THAT MOST APPLIES
  - - - For less than one month
      - For one to three months
      - For more than three months; please state approx. how many months:_____________________________________
- No, we do not use it

1. In your opinion, what are the BENEFITS of using the point of care rapid dengue test in your clinic? TICK ALL THE APPLY.

- Saves waiting time
- Helps case management
- Helps manage surges in dengue cases
- Helps gain more accurate diagnosis without lab work
- Keeps the resources used for diagnosis to a minimum
- Can’t say, don’t know enough about it yet
- Others (please specify):________________________________

1. What are some CHALLENGES you face using the point of care rapid dengue test in your clinic? TICK ALL THAT APPLY

- Will take up too much precious time training to staffs
- Inaccurate devise – I don’t trust it
- It puts too much work onto nursing staff
- Not cost effective
- No challenges
- I don’t feel I can comment
- Others (please specify):__________________________________

1. Please tell us about out your experience of using the point of care rapid test in your clinic, or if you haven’t used it how might such a test be implemented in your clinic?

On the current (2013-2014) spike in Dengue cases

1. During the current spike in dengue cases, please tell us what you did to manage the surge in dengue cases:
2. Singapore experienced large dengue spikes in the year 2004/05 and 2007. Were you practicing in Singapore during any of those periods?

- Yes
- No

1. (For all participants) IF you were practicing in Singapore during any of those dengue outbreaks, can you tell us the main ways in which your clinical management practices might have changed since then?

**Thank you for your time and effort.**
