# Supplementary material for: Assessing changes in knowledge, attitude and practices on dengue diagnosis and management among primary care physicians after the largest dengue epidemic in Singapore
Source: BMC Infect Dis. 2017 Jun 15;17:428. doi: 10.1186/s12879-017-2525-3 (PMC5472871; doi:10.1186/s12879-017-2525-3)
Supplement: Supplementary file 3 — The clinical scenarios used to assess the level of clinical management. Data on the three clinical scenarios surveyed to assess the level of clinical management. (DOC 55 kb) [file 12879_2017_2525_MOESM3_ESM.doc]

Additional File 3

Title: The clinical scenarios used to assess the level of clinical management.

Description: Data on the three clinical scenarios surveyed to assess the level of clinical management.

1. Clinical Scenario I: The full blood count of a patient at day 2 of fever revealed total white cells of 8,700/ul (Poly 75%, lymph 15%, monocyte 5%, others 5%), hemoglobin of 15g/dl. By the look of the full blood count alone, I can conclude that dengue is an unlikely diagnosis.

- Yes – 13.7%
- **No – 76% (Correct Choice)**
- Not sure – 10.3%

1. Clinical Scenario II: During the entire course of dengue, the critical period denotes the period when patients are having high fever and look ill.

- Yes – 16.4%
- **No – 79.8% (Correct Choice)**
- Not sure – 3.8%

1. Clinical Scenario III: The minimum and maximum hematocrit readings of a confirmed dengue patient were 42% and 51% respectively, what was the percentage change of hematocrit?

- 17.6% - 7%
- **21.4% - 51% (Correct Choice)**
- 9% - 18.5%
- Not sure – 23.4%
