# Supplementary material for: Assessing changes in knowledge, attitude and practices on dengue diagnosis and management among primary care physicians after the largest dengue epidemic in Singapore
Source: BMC Infect Dis. 2017 Jun 15;17:428. doi: 10.1186/s12879-017-2525-3 (PMC5472871; doi:10.1186/s12879-017-2525-3)
Supplement: Supplementary file 4 — Perceptions of PCPs on POCT and clinical management experience during 2013 outbreak. Data on the perceptions of PCPs who do not have any experience on POCT and the management experience and changes during and after the 2013 outbreak. (DOCX 23 kb) [file 12879_2017_2525_MOESM4_ESM.docx]

**Additional File 4**

**Table S1. Perceptions of PCPs who do not have any experience on POCT**

| **Themes** | **Frequency** | **Quotes** |
| --- | --- | --- |
| - **Concerns:** |  |  |
| Cost | 21 | *“Kits have to be cheap and accessible.”* |
| Accuracy | 19 | *“Need to know sensitivity and specificity of test.”* |
| Need to be free or highly subsidized | 2 | *“Enough free samples or highly subsidized tests”* |
| Lack of official report | 1 | *“No formal test report was given to patient.”* |
| - **Cannot be implemented due to the following:** |  |  |
| Overwhelmed workload | 17 | *“Labour shortage is an acute problem in most GP solo practices.”*  *“The test would be done by me personally as my staff is already very busy. So it will be slowing me down”* |
| No impact on patient’s management | 5 | *“Decision to admit patient still based on clinical parameters and FBC.”* |
| Other free tests available | 3 | *“Not necessary as the EHI diagnostics provides Free bloods test for suspected Dengue cases.”* |
| Unsure/Not decided by doctor | 17 | *“Not sure at this moment”*  *“Decided by Head since it is a polyclinic”* |
| - **Other requirements** |  |  |
| Training and sample kits are needed | 14 | *“Formal training session with demo.”* |
| Ease of use | 7 | *“Depends on ease of use and time taken”* |
| Availability | 4 | *“Test kits will be used if available.”* |
| Consignment/expiry kit | 4 | *“If the test is done on consignment basis, clinic may opt for it. Otherwise there is not enough patient to support the test kit.”*  *“Expiry test kits would be a concern then.”* |
| Portable | 3 | *“It must be portable, reliable and reasonably cheap.”* |
| - **Positive attitude** |  |  |
| Improve patient's management | 5 | *“The rapid test kit will improve management of febrile patients.”* |
| Keen | 5 | *“Not used, keen though.”* |

**Table S2. Management experience of Dengue spike in 2013 and 2014.**

| **Themes** | **Frequency** | **Quotes** |
| --- | --- | --- |
| **Surge management** |  |  |
| - More vigilant | 50 | *“Lower Threshold of suspicion for Dengue.”* |
| - Manageable/no surge seen | 38 | *“Managed Easily”*  *“Not significant surge in clinic”*  *“Unable to comment as most of the patients I see are chronic patients”* |
| - Practice as normal | 37 | *“No Changes in management of patients”* |
| - Follow guidelines/Wait for instructions | 6 | *“I do follow the clinical guideline provided by MOH.”*  *“Locum at polyclinic. Then usually wait for instructions from HQ.”* |
| - Prioritizing patients/see more patients | 5 | *“Patient with fever were separated from the rest of the patients, for earlier review.”*  *“Work harder. See more patients.”* |
| **Diagnosis management** |  |  |
| - Dengue test (duo kit, IgG/M, NS1) | 67 | *“All suspected cases were advised to do FBC and Dengue test.”*  *“Blood test for FBC +NS1/IgG/IgM were sent to lab on urgent basic”* |
| - Full Blood Count | 46 | *“To do FBC and Dengue serology in suspected cases”* |
| - Examine history and clinical symptoms | 7 | *“accurate clinical triage + screening.”* |
| - Notify cases/location | 9 | *“Notification of case to MOH”*  *“Try to ascertain geographic location of the cases. Notify cases.”* |
| Patients' management |  |  |
| - Close monitoring/FBC monitoring | 62 | *“Monitor patients daily if diagnosis is made.”* |
| - Patient education/insect repellent | 33 | *“Patient education to watch out for worsening symptoms for review if so”* |
| - Make referral | 23 | *“Refer to A/E where clinical/haematological criteria met”* |

**Table S3. Changes in Dengue management**

| **Themes** | **Frequency** | **Quotes** |
| --- | --- | --- |
| Better Dengue test available | 57 | *“Blood diagnostics efficiency has improved.”* |
| Better care provided | 24 | *“I think management of cases better now as we are more aware and confident of treating most Dengue cases….”*  *“Improved.”* |
| Better patients' awareness | 21 | *“More awareness nowadays for patients as well.”* |
| Stricter criteria for admission now | 21 | *“Lower platelet levels for hospital referrals.”* |
| More were managed as outpatients | 5 | *“Less hospital admission.”* |
| Better doctors’ awareness | 11 | *“GPs now more aware & more likely to do early detection.”* |
| Clearer guidelines | 5 | *“there are clearer and better clinical guidelines.”* |
| Location of Dengue cases | 4 | *“Availability and media hot spot announcement”* |
| No change/not sure | 18 | *“No Change.”* |
