# Supplementary material for: Assessing changes in knowledge, attitude and practices on dengue diagnosis and management among primary care physicians after the largest dengue epidemic in Singapore
Source: BMC Infect Dis. 2017 Jun 15;17:428. doi: 10.1186/s12879-017-2525-3 (PMC5472871; doi:10.1186/s12879-017-2525-3)
Supplement: Supplementary file 5 [file 12879_2017_2525_MOESM2_ESM.odt]

Additional File 2

Title: The awareness of the dengue warning signs among primary care physicians 

Description: Data on dengue warning signs recommended by the World Health Organization (WHO) that the participants are aware of.

| Q10 – WHO Dengue Warning signs | N (%) |
| --- | --- |
| Bleeding/Mucosal bleeding | 139 (55.6) |
| Abdominal pain or tenderness | 97 (38.8) |
| Drowsiness, fatigue, lethargy, breathlessness | 44 (17.6) |
| Persistent vomiting | 11 (4.4) |
| Clinical fluid accumulation | 10 (4) |
| HCT rise and PLT drop | 9 (3.6) |
| Hepatomegaly | 1 (0.4) |
| HCT rise # | 56 (22.4) |
| PLT drop # | 40 (16) |
| Vomiting # | 28 (11.2) |
| Total participants who have heard of dengue warning signs | 250 |

#- Inaccurate response
